# 

**Published:** 1898-09

**Authors:** 


### CONTENTS:

### SELECTIONS.

Article I.—Responsibility in Administering an Anesthetic........ 193
" II.—Clinical Report on Method of Operation. Dr. H. C. Register.................................................. 195
" III.—The Mouth is the Via Natura for the Entrance of Disease. M. V. Ball, M. D ........................... 198
" IV.—The Preparation of the Mouth for a Denture. Robert H. Nones, D. D. S........................................ 204
" V.—Using Soft Gold. D. Benj. Lord.......................... 210
" VI.—Bites and Some Methods of Taking Them. J. Mahoney 214
" VII.—Prosthetic Dentistry. Dr. Alvin J. Hovey............ 222
" VIII.—Dental Technic. Dr. J. Quincy Byram................ 225
" IX.—On Amalgam Fillings. Thomas Fletcher, F. C. S..... 231
" X.—The First Subscribers to the First Dental Journal.... 232

### MONTHLY SUMMARY.

The Patent Bill.................................................. 238

Destruction of Pulp and Gold Crown by Mercuric Action Within Amalgam.......................................................... 238

Tin.................................................................. 239

Shrinkage of Amalgams........................................... 240

Getting the Bite .................................................. 240

